# Life Preservation from a Business Point of View

**Published:** 1890-10-25

**Authors:** 


### Life Preservation from a Business Point of View.

It has often been more or less seriously suggested that the relations between doctors and patients in Europe and America are inexpedient from a business point of view, and that a better system is that of some Oriental countries where the physician is paid an annual fee so long as his client remains well, but forfeits it while he is sick. No attempt has been made to carry out this plan in Western countries, but the general idea that the physician's pay should be in the form of a regular annual sum for each person under his care, no matter whether they are sick or well, and that it should not be to his interest to have the illness of any of his patients prolonged, is by no means an uncommon one, and is acted on in the provident dispensary system. Attempts have also been made to establish life insurance companies on the basis of a particular system of medical practice, viz., homœopathy, thus giving a practical pecuniary evidence of faith that persons treated by doctors of this sect are less likely to die than others.

We do not remember, however, to have seen before such a scheme as is elaborated in a recent work entitled "La Conservation de la vie Humaine," édition albaverse, by A. Pichou.

An "édition albiverse" is a new name rather than a new thing, for it simply means that each alternate page is left blank white paper for notes and observations by the reader.

The scheme proposed is for the creation of a business corporation, company, or society, which undertakes to furnish its clients, subscribers, or members with medical advice and treatment during their lives, and to pay to their heirs a certain sum of money when they die.

The constitution and bye-laws for such a society, to cover France, Algeria, and Tunis, are given at length, and we select a few sections which will give our own joint stock company promoters some ideas as to how their scheme for Greater Britain should be drawn up.

The capital stock is to be 200,000,000 francs, divided into 400,000 shares of 500 francs per share at par, one-fourth to be paid down at the time of subscription, the remainder as called for by the council of administration on one month's notice.

The council of administration is composed of fifteen persons elected by the shareholders. Each of these must own at least one hundred shares in the company during the time he serves. Three councillors are elected each year, the whole term of service for which each is elected being five years.

Under the direction of this council there are to be established in each of the twenty arrondissements, or districts, of Paris, in each canton in France, and in the principal localities of Algeria and Tunisia, a medical centre, including a hospital and a place of meeting, the total number of such centres being fixed at 3,000.

Each hospital is to contain a number of single rooms for patients, a pharmacy, lodging for the pharmacist, and baths. The central place of meeting, or conservatory, as it is called, is substantially a dispensary.

The scale of fees fixed is as follows:—

1. Subscription for one person, giving the right to medical attendance and medicines from the company for one year, 15 francs. In addition to this, if the sick person is treated in one of the hospitals of the company he is to pay three francs per day.

2. The annual payment for insurance to the amount of 1 000 francs, to be paid by the company on the death of the insurer, is fixed according to the age at which the payments commence, as is usual in life insurance companies. At the age of from seven to eight years the annual payment would be 11 francs 55 centimes; at from 15 to 16 years, 13 francs; from 36 to 37, 21 francs, and so on. No insurance can be made for more than 1,000 francs.

The subscriptions must be paid in advance, and the company has the right to refuse to accept any particular person as a subscriber.

All subscribers are to be visited by the medical officers at least twice a-year, and their state of health and sanitary surroundings carefully inquired into.

It is proposed that the company shall have certain special relations with the State, and that its officials in each district or commune shall be the local health officers.

Every principal centre is to have a resident medical officer, who must be a doctor of medicine, a pharmacist of the first class, and a midwife. Other details are given at great length, together with the form of a law governing the whole business, but we have probably stated enough to give an idea of the scheme.

It will be seen that it is by no means a purely voluntary business corporation that is proposed; in fact, after outlining the plan of such a society in the first part of the work, it is declared that it cannot pay its expenses from its fees unless the State gives it certain work to do. There is, of course, not the least probability that any such scheme of organization will be carried out in the near future, for it would contravene the interests not only of the great mass of the medical profession, but of many local officials; but it is interesting as a sign of the times, and as a more than usually definite statement of the ideas of a certain class of reformers.

